# Changes in C-reactive protein, neopterin and lactoferrin differ after conservative and surgical weight loss in individuals with morbid obesity

**DOI:** 10.1038/s41598-019-54107-z

**Published:** 2019-11-27

**Authors:** Martin Aasbrenn, Per G. Farup, Vibeke Videm

**Affiliations:** 1 Department of Surgery, Innlandet Hospital Trust, Gjøvik, Norway; 2 Department of Medicine and Geriatrics, Bispebjerg and Frederiksberg Hospital, Copenhagen, Denmark; 3 Department of Research, Innlandet Hospital Trust, Brumunddal, Norway; 4 Department of Clinical and Molecular Medicine, Faculty of Medicine and Health Sciences, NTNU – Norwegian University of Science and Technology, Trondheim, Norway; 5 Department of Immunology and Transfusion Medicine, St. Olavs University Hospital, Trondheim, Norway

**Keywords:** Prognostic markers, Obesity

## Abstract

C-reactive protein, neopterin and lactoferrin are biomarkers of atherosclerotic disease. We aimed to assess changes in these biomarkers after conservative and surgical weight loss interventions in individuals with morbid obesity, to evaluate associations between biomarker changes and changes in body mass index and HbA1c, and to study associations between changes in the biomarkers. C-reactive protein, neopterin and lactoferrin were measured before and after conservative weight loss intervention and bariatric surgery. Data were analysed with mixed models. 137 individuals (mean age 43 years) were included. Body mass index decreased from 42.1 kg/m^2^ to 38.9 kg/m^2^ after the conservative intervention, and further to 30.5 kg/m^2^ after bariatric surgery. All biomarkers decreased after the conservative weight loss intervention. C-reactive protein and lactoferrin continued to decrease following bariatric surgery whereas neopterin remained stable. After adjustments for change in body mass index and HbA1c, all biomarkers decreased significantly after the conservative weight loss intervention, whereas none changed after bariatric surgery. There were no consistent correlations between changes in C-reactive protein, neopterin and lactoferrin. In conclusion, biomarkers of atherosclerosis decreased after weight loss interventions but had different trajectories. Neopterin, a marker related to atherosclerotic plaque stability, decreased after conservative weight loss but not following bariatric surgery.

## Introduction

Obesity is associated with many comorbid conditions and increased mortality; 4 million deaths worldwide in 2017 were attributed to excess body weight^1–3^. Systemic low-grade inflammation with increased blood levels of biomarkers of inflammation is common in individuals with obesity^3,4^. Large weight loss in individuals with morbid obesity (MO) reduces both low-grade inflammation and the risk of cardiovascular disease, cancer and death^4,5^. Whether causal relations exist between the biomarkers of inflammation and the main comorbidities of obesity is still uncertain^6^.

The inflammatory process is complex and involves many different cell types and mediators^7^, including C-reactive protein (CRP), neopterin, and lactoferrin. CRP is a general marker of inflammation that is widely used clinically. CRP is often used as a marker of acute bacterial infection but is also increased in chronic inflammatory conditions such as rheumatic disorders. Elevations in CRP concentrations are usually much lower in conditions with chronic low-grade inflammation like morbid obesity or atherosclerosis^2,4,8^. CRP is stimulated by the adipokine IL-6, and CRP levels are reduced after weight loss^4^. Less is known about changes in other biomarkers of inflammation such as neopterin and lactoferrin after weight loss.

Neopterin is released from activated macrophages during various forms of acute or chronic inflammation. In cardiovascular medicine it has been used as a biomarker of atherosclerotic plaque stability^9,10^ even if it is not specific for this outcome. Neopterin is also inversely associated with endothelial function measured as flow-mediated dilation^11,12^.

Lactoferrin is released from activated neutrophils with functions including antimicrobial effects, immunomodulatory properties, and regenerative properties^13^. Macrophages are activated by lactoferrin^13^, which may be of importance in the pathogenesis of cardiovascular disease. Lactoferrin concentrations were higher in individuals with significant coronary artery stenosis than in those without^14^. The link between neutrophil and macrophage activation may also explain our previous finding that higher concentrations of lactoferrin predicted fatal ischemic heart disease in long-term follow-up in individuals with diabetes type 2^15^.

The hypotheses for the study were that because the included biomarkers represent different aspects of inflammation, their changes following conservative and surgical weight loss interventions would not necessarily follow the same patterns and that the changes could be different in the two treatment periods. Furthermore, we hypothesized that the biomarker changes may be associated with changes in BMI or HbA1c, which were considered as available proxies of changes in adiposity and glucose tolerance, respectively.

The present study had the following aims: (1) To assess changes in the biomarkers CRP, lactoferrin, and neopterin after conservative and surgically induced weight loss in individuals with MO and to compare the changes in the two treatment periods (2) To assess whether the biomarker changes in the two treatment periods were associated with changes in BMI and HbA1c in multivariable analysis, and to compare the associations in the two treatment periods. (3) To study the associations between changes in CRP, neopterin and lactoferrin after weight loss.

## Methods

### Design and participants

Adults referred to the obesity centre at Innlandet Hospital Trust in South-Eastern Norway from December 2012 through September 2014 were eligible to this prospective study^16^. In the present sub-study, individuals with blood samples analysed for biomarkers and with complete data on weight, height, physical activity, and comorbidities comprising diabetes, hypertension and chronic obstructive pulmonary disease (COPD), were included. Information from three visits was used; a first visit to the hospital, a second visit around 5 months later after the conservative weight loss intervention and before the bariatric surgery, and a third visit 6 month after surgery.

### Inclusion and exclusion criteria

Inclusion criteria were age between 18 and 65 years and MO, defined as BMI > 40 kg/m^2^ or BMI > 35 kg/m^2^ with obesity-related comorbidity^17^. Exclusion criteria were major psychiatric disorders, drug or alcohol addiction, and serious somatic disorders not judged as obesity-related, i.e. conditions that would preclude usual weight loss treatment at the clinic. The participants thus represented the usual selection of individuals treated at the clinic and were receiving standard care for any comorbidities. They were asked to report any ongoing illnesses, and blood sampling was performed during routine visits when they reported to be well.

### The first visit

At the first study visit, clinical data were registered, and blood samples were drawn. Information about comorbidity was provided by the participant on a case report form. This information was reviewed by a physician with full access to the hospital records.

### The conservative weight loss program

The participants underwent a five month long conservative weight loss intervention with changes in diet and physical activity^18^. This intervention was performed in groups and was based on a series of outpatient visits. It was comparable to weight loss programs used in other Norwegian hospitals in this time period^19^ with some small local changes due to availability of personnel. All included participants started with three hour-long consultations with three health professionals; a nurse, a nutritionist and a physician, which included personalized advice on diet and physical activity. These consultations were usually scheduled in three separate weeks to give the participant time to implement suggested changes. Subsequently, all participants were enrolled in a patient group with weekly four-hour meetings during seven consecutive weeks. The meetings included group counseling together with other participants with morbid obesity, and were led by nurses specialized in obesity, nutritionists, surgeons and a psychologist.

The dietary advice was based on reduction of total energy intake and choice of food rich in micronutrients^18,20^. The participants were recommended to eat more fiber and protein, less sugar and fat, and to distribute their food intake into 4–6 meals each day with 2–4 hour intervals between meals. Advice on specific dishes was individualized by a nutritionist based on the individual’s former diet and food preferences. 21 days before the second study visit the participants were advised to follow a “crisp bread diet” containing 1000 kcal/day. The recommendations for this diet was intake of six pieces of crisp bread with low-fat high-protein topping (cheese, meat or fish), 4.5 dl of low-fat milk, a small dinner plate (meat or fish with vegetables), free amounts of vegetables (not corn, olives, or avocados) and free amounts of beverages without calorie content (preferentially above 2 litres). As an alternative to the crisp bread diet, the participants could use meal replacement powder giving 800–900 kcal with vegetables. All participants were told that acceptance to the clinic’s public, free-of charge bariatric surgery program partly depended on their adherence to the given advice.

### The second visit, before surgery

The second study visit took place after the conservative weight loss program, on average 23 weeks after the first visit, one week before planned bariatric surgery. Clinical data were registered, and blood samples were drawn.

### The surgical procedure

The participants subsequently underwent bariatric surgery, either as laparoscopic Roux-en-Y Gastric Bypass^21^ or laparoscopic gastric sleeve^22^.

### The follow-up visit six months after surgery

The third visit was a postoperative follow-up at the outpatient clinic for MO about six months after bariatric surgery, on average 49 weeks after the first visit. Clinical data were registered, and blood samples were drawn.

### Variables

The present study used a subset of the variables in the main study. Age, sex, body mass index (BMI, calculated as the weight in kilograms divided by the square of the height in meters), comorbidities (chronic obstructive pulmonary disease, diabetes, hypothyroidism, previous myocardial infarction, angina, previous stroke, hypertension), total cholesterol, LDL cholesterol, HDL cholesterol and HbA1c were registered in addition to serum concentrations of the inflammatory biomarkers CRP, neopterin and lactoferrin.

CRP and HbA1c were analysed at Innlandet Hospital Trust Gjøvik with a Cobas c501 instrument with the reagents CRPL3 and Tina-quant HbA1C (Roche Diagnostics GmbH, Mannheim, Germany). Reference values were <5 mg/L for CRP and 4.0–6.0% for HbA1c. Neopterin was quantified using a commercial enzyme immunoassay (Genway Biotech, San Diego, USA). Lactoferrin was analysed using an enzyme immunoassay as previously reported^23^. For these assays, neopterin values above 10 nmol/L are evaluated as pathological. In a group of 302 blood donors, lactoferrin values were below <250 ug/L (V Videm, unpublished data). Total cholesterol, low- and high-density lipoprotein (LDL and HDL, respectively) cholesterol were analysed using standard methods at Innlandet Hospital Trust Gjøvik.

### Statistical analysis

Data are given as mean (standard deviation – SD) or number (percentage). Statistical analysis was performed using mixed models. The time variable was coded as time 0 (first visit), 23 weeks (second visit) and 49 weeks (follow-up visit after bariatric surgery). Mixed models allow for simultaneous analysis of the three repeated (i.e. correlated) measurements in each individual and direct comparison of the changes after conservative weight loss with the changes following bariatric surgery. It also allows for individual trajectories of the variables in each individual, and different numbers of participants included at each time point due to attrition during follow-up. These are realistic assumptions for biomarkers of inflammation, which show large individual variations, and weight-loss interventions, where it is unrealistic that all participants will complete all steps of treatment.

Data were first analysed in univariate models with CRP, neopterin, lactoferrin, BMI, or HbA1c as the dependent variable, in order to investigate the observed values and changes after the two weight loss interventions. Each biomarker was thereafter analysed in a multivariable model including BMI and HbA1c to investigate associations of the biomarker changes with changes in adiposity and glucose tolerance, also including adjustments for age and sex to avoid confounding due to increased low-grade inflammation with older age or to sex-related differences. To evaluate whether the associations between each biomarker and BMI or HbA1c were different after the conservative and the surgical weight loss interventions, interaction terms with time were evaluated. Model fit was assessed using residual plots.

For an analysis of bivariate associations, Pearson’s correlation coefficients were calculated between changes in CRP, neopterin and lactoferrin as well as between changes in each biomarker and changes in BMI or HbA1c after the two weight loss interventions. This method does not take within-person correlation into account. Therefore, unadjusted mixed models including one biomarker as the dependent variable and another as the independent variable as well as an interaction term with time were also analysed, thus accounting for within-person correlation. Likewise, unadjusted mixed models including each biomarker as the dependent variable and either BMI or HbA1c as the independent variable and an interaction term with time were analysed to assess unadjusted associations between changes in each biomarker and changes in adiposity or glucose tolerance. Overall association in each of these models was evaluated using the between-person Snijders/Bosker R2 for mixed models. P-values < 0.05 were considered significant, and all tests were two-sided. Stata (v15.1, College Station, Texas, USA) was used for all analyses.

To assess the robustness of the findings in the analyses to investigate associations of the biomarker changes with changes in BMI and HbA1c, we performed sensitivity analyses with additional adjustment for smoking (coded as ever, former or present smoker) or gastric sleeve vs. gastric bypass operation. We also analysed models where body weight was included instead of BMI.

### Ethics

The study was approved by the Regional Committee for Medical and Health Research Ethics South East Norway (reference 2012/966) and conducted in accordance with the Declaration of Helsinki. Written informed consent was obtained from all participants included in the study.

## Results

A total of 137 participants of which 135 had complete baseline data for each of the three biomarkers (78% females) with a mean age of 43 years and a mean BMI of 42 kg/m^2^ were included (Table 1). The levels of inflammatory biomarkers were slightly increased at inclusion. A third of the participants had hypertension and a fifth of the participants had diabetes mellitus, but few of the included participants had developed atherosclerotic disorders as acute myocardial infarction or stroke.

**Table 1:** Baseline characteristics of the participants.

Variable	
Age (years)	43 (9)
Female sex (%)	105 (78)
Height (cm)	171 (9)
Weight (kg)	124 (19)
Chronic obstructive pulmonary disease	3 (2)
Diabetes	24 (18)
Hypothyroidism	19 (14)
Previous myocardial infarction	2 (1)
Angina	1 (1)
Previous stroke	4 (3)
Hypertension	44 (33)
Smoking never/former/present	56 (41)/55 (41)/24 (18)
Total cholesterol (mmol/L)	5.1 (0.9)
LDL cholesterol (mmol/L)	3.3 (0.9)
HDL cholesterol (mmol/L)	1.2 (0.3)

Data are given as mean (standard deviation) or number (percentage).

**Figure 1:**
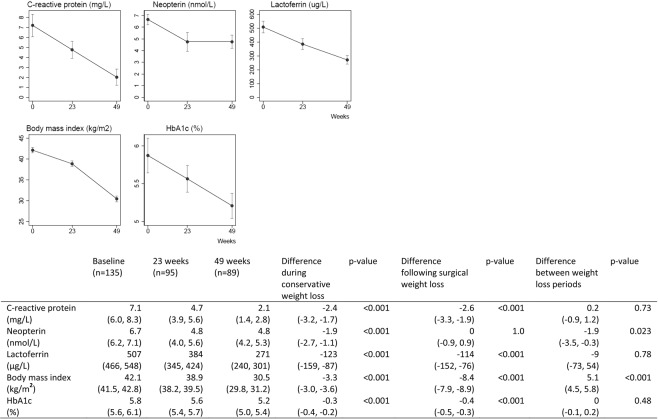
Biomarkers of inflammation, body mass index and HbA1c (observed values). Data are given as means with 95% confidence intervals. Statistical testing with mixed models.

**Figure 2:**
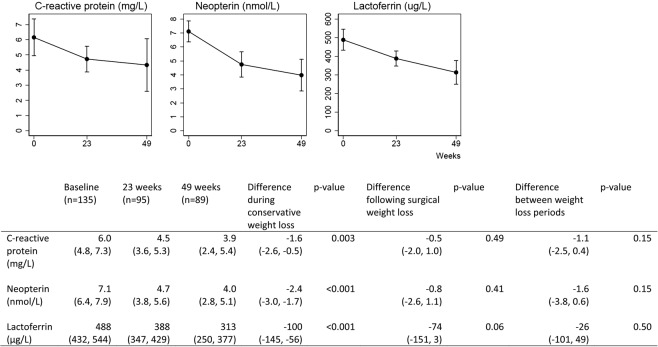
Biomarkers of inflammation adjusted for sex, age and changing body mass index and HbA1c. Data are given as means with 95% confidence intervals. Statistical testing with mixed models.

BMI was reduced from 42.1 kg/m^2^ to 38.9 kg/m^2^ after the conservative weight loss intervention, and further to 30.5 kg/m^2^ after the surgical weight loss intervention. After the conservative weight loss intervention, blood levels of CRP, neopterin and lactoferrin had decreased significantly (Fig. 1). After the surgical weight loss intervention, we observed a further decrease in CRP and lactoferrin, but no change in neopterin (Fig. 1). Glucose tolerance measured as HbA1c was improved both after the conservative and after the surgical weight loss intervention. After adjustment for changes in weight and glucose tolerance, we still found a significant reduction in CRP, neopterin and lactoferrin after the conservative weight loss intervention, but no significant changes in CRP, neopterin or lactoferrin after the surgical weight loss intervention (Fig. 2). The changes in CRP were significantly associated with changes in BMI in the multivariable models (Table 2), whereas the changes in the other biomarkers were neither associated with changes in BMI nor HbA1c. We did not find consistent correlations between changes in CRP, neopterin and lactoferrin after the two weight loss periods in the bivariate analysis (Table 3).

**Table 2:** Associations between changes in biomarkers and changes in other variables^a^.

	Dependent variable		
	C-reactive protein	Neopterin	Lactoferrin
**Main multivariable models**			
Body mass index	0.22 (0.04–0.40) p = 0.016	−0.11 (−0.23, 0.02) p = 0.10	5.98 (−1.92, 13.88) p = 0.14
HbA1c	0.63 (−0.14, 1.39) p = 0.11	−0.05 (−0.38, 0.28) p = 0.76	−16.18 (−37.76, 5.40) p = 0.14
Sex	−1.62 (−3.23, −0.01) p = 0.048	0.48 (−0.95, 1.91) p = 0.51	−38.26 (−98.66, 22.14), p = 0.21
Age	−0.07 (−0.15, 0.00) p = 0.07	−0.02 (−0.06, 0.03) p = 0.48	0.23 (−3.80, 4.27) p = 0.91
**Multivariable interaction models**^b^			
Body mass index * weight loss period			
Body mass index * Conservative weight loss period Body mass index * Surgical weight loss period Overall interaction^c^	−0.17 (−0.44, 0.12), p = 0.27 –0.24 (−0.50, 0.02), p = 0.07 p = 0.12	−0.28 (−0.60, 0.04), p = 0.09 0.31 (−0.05, 0.67), p = 0.09 p = 0.21	−3.68 (−14.21, 6.86), p = 0.49 −1.14 (−10.78, 8.50), p = 0.82 p = 0.75
HbA1c * weight loss period			
HbA1c * Conservative weight loss period HbA1c * Surgical weight loss period Overall interaction^c^	0.04 (−0.58, 0.67), p=0.90 0.45 (−0.78, 1.68), p=0.47 p = 0.70	−0.12 (−0.64, 0.40), p = 0.65 0.92 (−0.02, 1.85), p = 0.054 p = 0.11	−0.61 (−37.47, 36.25), p = 0.97 34.65 (−2.40, 71.71), p = 0.07 p = 0.18

^a^Coefficients (95% CI) from multivariable mixed models.

^b^Coefficients from similar multivariable mixed models including additional interaction term.

^c^Gives comparison of differences in associations between weight loss interventions.

**Table 3:** Bivariate associations among changes in biomarkers and changes in body mass index or HbA1c.

Associations (dependent variable vs. independent variable)	Conservative weight loss period		Surgical weight loss period		Overall interaction term from mixed model^c^	R2 for mixed model^d^
	Pearson’s R^a^	Coefficient from mixed model^b^	Pearson’s R^a^	Coefficient from mixed model^b^		
C-reactive protein vs. neopterin	−0.02 p = 0.82	−0.25 (−0.70,0.21) p = 0.29	0.03 p = 0.81	0.27 (−0.06, 0.59) p = 0.11	p = 0.27	0.03
C-reactive protein vs. lactoferrin	0.10 p = 0.34	0.00 (−0.01, 0.00) p = 0.63	0.24 p = 0.03	0.00 (−0.01, 0.00) p = 0.69	p = 0.81	0.05
Neopterin vs. lactoferrin	−0.02 p = 0.84	0.00 (0.00, 0.01) p = 0.25	0.11 p = 0.34	0.00 (0.00, 0.00) P = 0.86	p = 0.41	0.09
C-reactive protein vs. body mass index	0.00 p = 0.98	−0.15 (−0.42, 0.12) p = 0.27	0.06 p = 0.63	−0.25 (0.00, 0.50) p = 0.053	p = 0.10	0.10
C-reactive protein vs. HbA1c	−0.06 p = 0.54	0.11 (−0.50, 0.72) p = 0.72	−0.10 p = 0.45	0.30 (−0.64, 1.23) p = 0.53	p = 0.67	0.06
Neopterin vs. body mass index	0.04 p = 0.70	−0.28 (−0.60, 0.04) p = 0.08	0.01 p = 0.96	0.30 (−0.05, 0.66) p = 0.09	p = 0.21	0.12
Neopterin vs. HbA1c	0.06 p = 0.58	−0.12 (−0.64, 0.39) p = 0.64	0.09 p = 0.45	0.67 (−0.16, 1.51) p = 0.12	p = 0.26	0.09
Lactoferrin vs. body mass index	0.14 p = 0.20	−1.83 (−12.81, 9.13) p = 0.74	0.17 p = 0.15	−2.59 (−12.29, 7.10) p = 0.60	p = 0.79	0.15
Lactoferrin vs. HbA1c	−0.18 p = 0.09	9.97 (−31.45, 51.38) p = 0.64	0.05 p = 0.66	17.08 (−28.89, 63.05) p = 0.47	p = 0.36	0.16

^a^Pearson’s linear correlation coefficient for variable changes during each period.

^b^Mixed model including interaction term with time. Coefficients are given for the interaction term, indicating association in each weight loss period.

^c^Gives comparison of differences in associations between weight loss interventions.

^d^Snijders/Bosker R2 for between-person variable in mixed model.

The sensitivity analyses showed that the associations of the biomarker changes with changes in BMI or HbA1c were essentially unaltered with adjustment for smoking or when considering the type of operative procedure. When body weight was used in the models instead of BMI, model fit was reduced. This finding indicates that changes in BMI rather than changes in weight were more closely related to the biomarker changes.

## Discussion

In this prospective study of individuals with MO, the levels of three biomarkers of inflammation were reduced in the blood after major weight loss. We observed different patterns after conservative and surgical weight loss, and different trajectories for CRP, neopterin, and lactoferrin.

After conservative weight loss, CRP, neopterin, and lactoferrin were all significantly reduced, both in unadjusted and adjusted analyses. Conservative weight loss interventions are associated with a modest long-term weight loss, but do nevertheless lead to reduced incidence of cardiovascular disorders and reduced mortality^24^. Low-grade inflammation is one of the probable mediators between lifestyle interventions and cardiovascular disease^6^. All the three biomarkers of inflammation examined in this study have been associated with cardiovascular events^6,15,25^. Interestingly, our study showed reduced low-grade inflammation after a conservative weight loss intervention also after adjustment for changes in BMI and glucose tolerance. Possible explanations could be the changes in diet, alterations in the signalling from the gut or changes in physical activity^3,24^. Earlier studies from our group have shown an association between the inflammatory load (neopterin and lactoferrin levels) and coronary artery disease^15,25^. Neopterin is associated with the stability of atherosclerotic plaques^10^, and in this regard it is notable that neopterin was significantly improved after a conservative weight loss intervention. However, the assayed biomarkers are not specific to atherosclerosis or other single endpoints.

The pattern of change after a surgical weight loss intervention was different and did not depend on the operative method (gastric sleeve vs. gastric bypass). The amount of excess weight lost after the intervention was much larger than the amount lost after the conservative weight loss intervention. However, the reductions in CRP and lactoferrin were not larger, and neopterin did not change after the surgical weight loss intervention. After adjustment for changes in glucose tolerance and BMI, the changes in inflammation after surgical weight loss were not significant. The study design precludes drawing of conclusions regarding precise mechanisms for the observed biomarker changes. The fact that models including BMI rather than weight changes showed better fit, may indicate that the mechanisms are not induced by weight changes alone but are more strongly related to differences in adiposity. Changes in low-grade inflammation after obesity surgery might have a different aetiology than changes after conservative weight loss interventions. These issues need further investigation in studies with different designs.

Reduction of mortality, cancer, and cardiovascular disorders after obesity surgery is well known and usually cited as the main reason for performing obesity surgery^5^. The reduction in cardiovascular disease after surgery is probably not only due to the surgery-induced anatomical changes; most individuals who undergo obesity surgery also implement major lifestyle changes before and after surgery. That conservative weight loss has a higher impact than the surgical weight loss on neopterin, a marker of coronary artery instability is intriguing in this regard. The studies that show mortality reduction after bariatric surgery are of high quality but not randomized^5,26^, whereas the randomized studies of surgery are smaller and only show effects on surrogate endpoints^27^.

Low-grade inflammation is often associated with measures of glucose tolerance and obesity in cross-sectional studies^4^. We did not identify strong correlations between changes in glucose tolerance or BMI and the changes in CRP, neopterin, and lactoferrin in this study. The low-grade inflammation in individuals with MO is more than a general marker of metabolic unhealth, and different markers are related to different specific pathophysiological entities^10,14^.

Weight loss is often recommended for individuals at high risk of obesity-related comorbidities, but the research on this topic contains many paradoxes^3,28^. As shown in our study, two different methods to lose weight do not have an equal impact on neopterin, and inclusion of more biomarkers of inflammation than CRP might be important in future studies of weight loss. The choice of weight loss intervention should probably be based on more factors than weight alone; including comorbidity load, body composition and possibly genetic markers and biomarkers. Different biomarkers of inflammation could be tested for clinical utility in this setting, and our study was not designed to investigate the usefulness of each biomarker on specific endpoints.

### Strengths and limitations

Strengths of the study include a relatively large cohort of participants, and adequate preanalytical handling of the tests. The study population was representative for all individuals referred to the clinic during the study period. The conservative weight loss program has been used with good experience in many obesity clinics in Norway^19^, and all bariatric surgery was performed by the same three experienced surgeons. A weakness is that all participants underwent their two periods of weight loss intervention in the same order with the conservative intervention first. It is therefore possible that the changes during the conservative weight loss period (e.g. the change in neopterin) were caused by this intervention coming first. Our findings should therefore ideally be re-examined in a study where participants are randomized to conservative or surgical weight loss interventions. Data regarding adherence to the conservative weight loss protocol were not available. The observed changes in weight during this period indicate that most participants have followed the advice to a degree giving measurable effects. The generalizability of the findings to individuals with a BMI under 35 kg/m^2^ is unknown. Detailed examinations of body composition might have been of interest to interpret the changes but were unfortunately not available. Even if it included 137 participants, the study might have false-negative findings due to the relatively large individual variation in concentrations of inflammatory markers. Long-term follow-up data, e.g. after 2 years, were not available.

## Conclusions

Three biomarkers of inflammation; CRP, neopterin and lactoferrin, were all significantly reduced in individuals with MO after a year with extensive weight loss. The changes were more prominent after conservative weight loss than after surgery-induced weight loss. Changes in CRP were associated with changes in BMI, whereas neopterin and lactoferrin did not show strong associations with HbA1c or BMI. Our data indicate that these inflammatory markers have different trajectories during weight loss and give information about different aspects of inflammation.

## Data Availability

Case report forms (CRFs) on paper were used for the collection of the clinical data, and all the CRFs are safely stored. The data were transferred manually to SPSS for statistical analyses. The data files are stored by Innlandet Hospital Trust, Brumunddal, Norway, on a server dedicated to research and with security according to the rules given by The Norwegian Data Protection Authority, P.O. Box 8177 Dep. NO-0034 Oslo, Norway. The data are available on request to the authors, but restrictions apply to the availability of these data according to Norwegian law so they are not publicly available.
